# F‐wave parameters and body mass index in carpal tunnel syndrome

**DOI:** 10.1002/brb3.2072

**Published:** 2021-02-15

**Authors:** Philip B. Adebayo, Rose E. Mwakabatika

**Affiliations:** ^1^ Neurology Section Department of Internal Medicine Aga Khan University Dar es Salaam Tanzania; ^2^ Clinical Neurophysiology Laboratory Aga Khan Hospital Dar es Salaam Tanzania

**Keywords:** carpal tunnel syndrome, F‐wave latency, F‐wave persistence, F‐wave latency, obesity, median–ulnar F‐wave latency difference

## Abstract

**Background:**

Severe carpal tunnel syndrome (CTS) readily lends itself to both clinical and electrophysiological recognition. The uncertainty sometimes is in identifying and quantifying motor involvement in mild and, perhaps, in moderate CTS. Our study aimed to evaluate F responses in mild and moderate CTS and determine the contribution of BMI to the F‐wave parameters.

**Methods:**

A retrospective review of the clinical and electrophysiological data of patients with CTS seen at the clinical neurophysiology laboratory of Aga Khan Hospital, Dar es Salaam, between 1 August 2017 and 31 July 2019 was retrieved. Carpal tunnel syndrome was graded according to the electrophysiological criteria of Padua. The F‐wave parameters of patients with mild‐to‐moderate CTS were analyzed and compared with asymptomatic controls.

**Result:**

We studied 91 hands. Twenty‐two hands were asymptomatic controls, 30 hands had mild CTS, and 39 hands had moderate CTS. Patients with moderate CTS were more obese (*p* =.011), had more females (*p* =.044), and were older (*p*= <0.001). F‐wave parameters were not convincingly different between mild and moderate CTS. F‐wave chronodispersion (*p* =.035) and F‐wave persistence (0.019) were significantly different between nonobese control and mild and moderate CTS. Median–ulnar F‐wave latency difference (FWLD) was significant between obese patients with mild CTS and moderate CTS scores (*p* =.017).

**Conclusion:**

Although a clear difference exists between F‐wave parameters in asymptomatic controls and those with CTS, the F‐wave study is inadequate in distinguishing mild and moderate CTS even in the context of BMI. Median–ulnar F‐wave latency difference (FWLD) appeared to be a promising discriminant parameter between obese patients with mild CTS and those with moderate CTS.

## Graphical abstract

We compared F wave parameters between normal individuals, patients with mild carpal tunnel syndrome and those with moderate carpal tunnel syndrome while considering the effect of obesity. F‐wave parameters clearly differs between asymptomatic controls and those with CTS. However, F‐waves study is inadequate in distinguishing mild and moderate CTS even when BMI is considered.

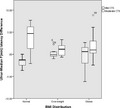

## 1 INTRODUCTION

F waves are late motor action potentials evoked in response to antidromic activation of motor neurons from the anterior horn cells. (Panayiotopoulos & Chroni, 1996) Since it assesses motor conduction along the whole length of a peripheral nerve, F‐wave analysis is classically valuable in evaluating generalized neuropathy and the proximal segments of a peripheral nerve while providing a glimpse into the integrity of central motor conduction. (Fisher, 2007) Although its value in assessing focal neuropathies is questionable, F‐wave anomalies have been demonstrated in focal neuropathies such as carpal tunnel syndrome (CTS). (Aalemdar, 2015; Aygül et al., 2014).

Anthropometric parameters such as height and BMI may influence nerve conduction parameters such as distal motor latency (DML) of the median nerve, and a prolonged F‐wave minimal (*F* (min)) latency of the tibial nerve lower sensory and mixed nerve amplitudes in healthy adults. (Buschbacher, 1998; Jerath & Shy, 2017) Aside from BMI, increasing age and the male gender correlated with decreasing amplitude and area values (Buschbacher, 1999) and the nerve conduction velocities. (Awang et al., 2007) Height is known to correlate with median F‐wave minimum latency directly. (Aalemdar, 2015; Puksa et al., 2003).

According to some authors, clinical severity and grading of CTS have been suggested to have a direct correlation with prolonged *F* (min) latency. (El et al., 2017) Other parameters from conventional electrodiagnostic studies such as median motor terminal latency index (m TLI), median motor residual latency (mRL), and median–ulnar F‐wave latency difference (F diff MU) have been suggested as potentially useful data. (Park et al., 2014) However, these parameters have not been proven to add any additional diagnostic benefit to the existing conventional electrodiagnostic studies for carpal tunnel syndrome. (Mondelli & Aretini, 2015).

Since severe CTS readily lends itself to both clinical and electrophysiological recognition, the uncertainty sometimes is in identifying and quantifying motor involvement in moderate and, perhaps, in mild CTS. This study aimed to evaluate F‐wave parameters in mild and moderate CTS while unraveling BMI's contribution to the F‐wave parameters.

## 2 METHODOLOGY

This study was a retrospective, comparative cross‐sectional study. We retrieved the clinical and electrophysiological data of patients referred for electrodiagnostic (EDX) evaluation of suspected CTS in our laboratory. The studies conducted between 1 August 2017 and 31 July 2019were assessed, and the data of patients with full F‐wave studies were recorded and coded for this analysis. This report is a subanalysis of F‐wave data from the larger cohort of patients with carpal tunnel syndrome from our laboratory. (Adebayo et al., August 2020) The ethical approval for the study was obtained from the scientific committee of the Aga Khan Hospital. Dar es Salaam.

### 2.1 Data Collection

We collected the records of patients from the laboratory logbook, electronic record system and the EMG machine. We included patients with signs or symptoms in keeping with CTS in at least two of digits I to III. All patients who had positive EDx findings and full F‐wave studies were included in this analysis. We excluded those with concurrent ulnar neuropathy and cervical radiculopathy. This study's controls were patients referred to our laboratory for EDX studies who were adjudged to have had musculoskeletal disorders after clinico‐electrographic evaluation. All the control subjects had standard electrophysiological studies. Patients with ulnar neuropathy or EDx features of cervical radiculopathy were excluded.

### 2.2 Electrodiagnostic Assessment

All EDX testing was performed by a trained EDx physician (PBA) using the Nihon Kohden (Tokyo, Japan), model number MEB‐2200/MEB 9,100 machine, and following the American Academy of Electrodiagnostic Medicine (AAEDM) guideline. We used disposable adhesive Ad/Agcl electrodes to record with the ground electrode placed between the stimulating and recording electrodes for all studies. We ensured supramaximal stimulation at a pulse duration set at 0.05/0.1 ms for sensory and mixed nerve stimulation and 0.2/0.5 ms for motor nerve stimulation. We set the filters at 20 Hz and 2 kHz for low‐ and high‐frequency settings, respectively, while sweep speed was set at 1 ms per division. The onset and peak latencies were measured for all the nerves. Amplitude was measured from the baseline to the peak of negative deflection. Sensory and motor conduction velocities were derived according to standards. We maintained the skin temperature at or above 32^0^C for all studies. Motor and sensory conduction studies of both the median and ulnar nerves were performed.

#### 2.2.1 Median sensory latency

The median nerve was antidromically stimulated at the wrist while recording over the index finger, the reference and active electrodes being 3cm apart. The cutoff value was set at 3.3ms for onset latency.

#### 2.2.2 Median motor distal latency

The median compound muscle action potential (CMAP) was recorded with the active electrode on the belly of abductor pollicis brevis (APB), while the reference electrode was placed just distal to the metacarpophalangeal joint even as the median nerve was stimulated at the wrist, and a 7‐cm interval was maintained between the electrodes. Whenever the results of the routine studies were equivocal, we performed the underlisted tests.

#### 2.2.3 Sensory median–ulnar difference

Antidromic stimulation of ulnar nerve was performed over the ulnar edge at the wrist while recording the sensory response on digit IV. Median nerve was stimulated as stated above with recording on digit IV. Peak latencies were measured for both nerves, and median–ulnar differences were derived. The cutoff value was set at 0.5 ms.

#### 2.2.4 Sensory median–radial difference

Radial nerve stimulation was delivered along the lateral forearm over the radial bone while recording over the first digit. Median nerve sensory response was also recorded on digit I upon stimulation at the wrist while maintaining a fixed distance of 12 cm between the recording and stimulating electrodes. Median–radial differences in peak latencies were derived. The cutoff value was set at 0.7 ms.

### 2.3 F‐wave recording

F‐wave recording was performed after motor conduction studies. The standard belly‐tendon recording technique was used, with the muscles relaxed and the recording cathode placed over the motor point. At least 16 supramaximal stimuli were applied over the proximal wrist creases at a distance 6cm from the abductor pollicis brevis (median nerve) and the abductor digiti minimi (ulnar nerve). The filter band pass was set at 3 Hz to 10 kHz, sweep speed was set at 5 milliseconds/division, and amplifier gain was set at 1 to 5 mV/division. The following F‐wave parameters were measured: F‐wave persistence; F‐wave chronodispersion; F min‐wave latency (F_min_); F‐wave maximum latency (F_max);_ F‐wave mean latency (F_mean_); F‐M latency difference; F‐wave duration; F‐wave amplitude; and M‐wave amplitude. Median–ulnar minimum latency difference and M/F amplitude ratio were derived accordingly.

### 2.4 Electrophysiological Severity

We staged CTS according to the five‐stage scale of progressive CTS electrophysiological severity by Padua (Supplementary Table 1). We excluded those with stages 4 and 5 (severe) from this analysis.

**TABLE 1:** Demographic and clinical characteristics of the study population

Variables	Total *n* = (91)	Controls *n* = 22	Mild CTS *n* = 30	Moderate CTS *n* = 39	Test statistics	*P* value
Age	47.19 ± 12.95	39.86 ± 8.09	43.50 ± 8.14	54.17 ± 14.77	*F* = 13.26	<0.001*
Gender: *n* (%) female	69 (75.8)	19 (20.9)	18 (19.8)	32 (46.4)	X = 6.25	0.044
Height (cm)	161.91 ± 9.09	164.65 ± 7.89	165.17 ± 9.25	157.85 ± 8.14	*F* = 7.86	0.001*
BMI	28.98 ± 4.91	26.84 ± 3.79	28.43 ± 5.29	30. 61 ± 4.71	*F* = 4.71	0.011*
Clinical symptom (*n* = 69)						
Symptom Only	17(24.6)	‐	14(46.7)	3 (7.7)	X = 5.135	0.077
Symptom + 1 sign	35(50.7)	‐	10 (33.3)	25 (64.1)		
Symptom + ≥ 2 signs	17(24.6)	‐	6 (20.0)	11 (28.2)		

### 2.5 Anthropometry

Height was measured to the nearest centimeter using a digital stadiometer (Pelstar, Illinois, USA). Weight in light clothing was measured to the nearest 0.1 kilogram. We calculated body mass index as a ratio of weight (kg) to height squared (m^2^). All measurements were made by the neurophysiology nurse (RM). We classified cases and controls as obese (BMI ≥ 30kg/m^2^) and nonobese (BMI < 30 kg/m^2^) according to the WHO (Obesity & Overweight, 2020).

### 2.6 Statistical analysis

Categorical variables were summarized as frequency and percentages, while group differences were analyzed using the Pearson chi‐square test. Continuous data were summarized as means ± standard deviation when normally distributed and median (interquartile range, IQR) for skewed data. Between‐group comparisons were made using independent Student *t* test and Mann–Whitney U test for normally distributed and skewed data, respectively. One‐way analysis of variance (ANOVA) with correction for multiple pairwise comparisons was conducted. Pearson correlation of BMI and height with F‐wave parameters was performed. The SPSS version 22.0 software (SPSS Inc., Chicago, IL, US) was used for all statistical analyses. A p‐value < 0.05 was considered statistically significant.

## 3 RESULT

A total of 91 hands belonging to 61 subjects were studied. We studied 69 hands of cases and 22 hands of control subjects. The mean age in years of the study population was 47.19 ± 12.95 years. The mean age ± *SD* of the cases was 49.53 ± 13.36, while that of the control was 39.86 ± 8.09. Sixty‐nine hands belonged to females (75.8%), while 22 (24.17) were males. The cohort consisted of twenty‐two (22) asymptomatic controls, thirty (30) with mild CTS, and thirty‐nine (39) with moderate CTS. Patients with moderate CTS were more obese (*p* =.011), had more females (*p* =.044), and were older (*p*= <0.001). Table 1 shows the demographic and clinical characteristics of the study sample.

Table 2 shows the F‐wave parameters between the control group, patients with mild CTS, and those with moderate CTS. Percentage F‐wave persistence was higher in controls compared with subjects with CTS (*p* = 0–018). Subjects with CTS had significantly longer chronodispersion compared to control individuals (*p* =.005). Control subjects had higher median nerve F‐wave mean amplitude and higher mean amplitude ratio at significant levels of 0.007 and 0.021, respectively. Minimum F‐wave latency was more delayed in the cohort with moderate CTS (*p* =.007) and so is the median–ulnar minimum latency difference (*p*= <0.001). The mean F‐wave amplitude was significantly higher among asymptomatic control (*p* =.007). All the other F‐wave parameters were not significantly different across the groups.

**TABLE 2:** F‐wave parameters between controls and mild and moderate CTS

F‐wave parameter	Median F‐wave persistence (%)	Median F‐wave chronodispersion (ms)	Median F‐wave minimal latency (ms)	Median F‐wave maximal latency (ms)	Median F‐wave mean latency (ms)	Median F‐M latency (ms)	Median F‐wave duration (ms)	Median F‐wave amplitude (uV)	Median M‐wave amplitude (mV)	Mean amplitude ratio (%)	Ulnar F‐wave minimal latency (ms)	Minimal ulnar‐median F‐wave latency difference (ms)
All	83.49 ± 21.13	2.57 ± 1.51	28.03 ± 2.83	30.23 ± 2.60	29.08 ± 2.52	23.81 ± 2.63	10.76 ± 2.67	0.44 ± 0.20	10.39 ± 5.01	5.04 ± 2.85	27.29 2.27	−0.64 2.48
Controls (*n* = 22)	95.77 ± 5.91	1.42 ± 0.94	27.62 ± 2.07	29.05 ± 1.90	28.33 ± 1.94	24.22 ± 2.03	9.54 ± 2.57	0.56 ± 0.27	9.32 ± 3.05	6.44 ± 3.39	27.59 2.45	−0.03 2.42
Mild CTS (*n* = 30)	86.30 ± 18.87	2.64 ± 1.61	26.92 ± 2.19	29.57 ± 2.21	28.25 ± 2.05	23.38 ± 2.62	11.02 ± 2.45	0.43 ± 0.16	11.25 ± 4.90	4.31 ± 1.78	27.33 2.52	0.41 1.94
Moderate CTS(*n* = 39)	81.33 ± 22.71	2.51 ± 1.45	28.89 ± 2.99	31.40 ± 2.79	30.14 ± 2.80	24.13 ± 2.63	10.56 ± 2.83	0.39 ± 0.17	10.33 ± 5.89	4.81 ± 2.98	27.10 1.98	−1.78 ± 2.45
Statistics	4.209	5.592	5.222	8.33	6.79	1.011	2.010	5.334	0.956	4.031	0.334	8.804
P‐value	0.018*	0.005*	0.007*	<0.001*	0.002*	0.368	0.140	0.007*	0.388	0.021*	0.717	<0.001*
Eta	0.295	0.336	0.326	3.99	0.366	0.150	0.209	0.329	0.146	0.290	0.087	0.408

Note:

Negative (‐) number = median nerve values are less than those of the ulnar nerve.

* significant value.

**FIGURE 1:**
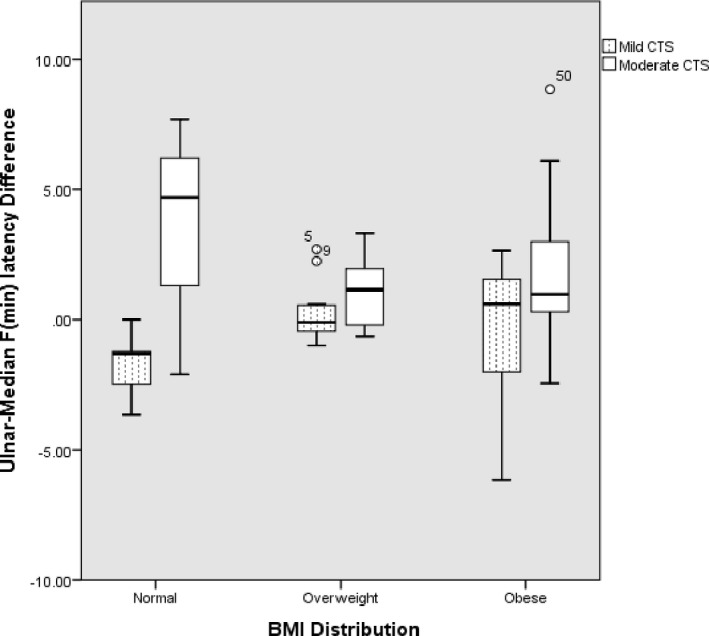
Median–ulnar F‐wave latency difference (FWLD) in normal, overweight, and obese patients

**FIGURE 2:**
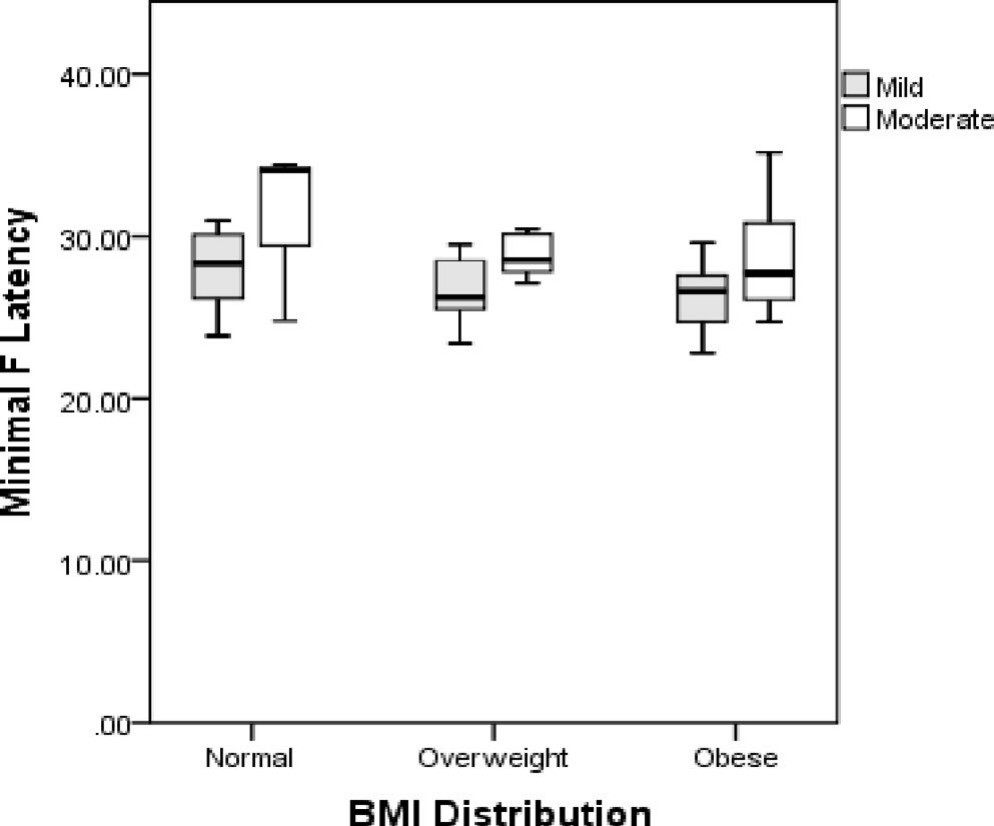
Median F‐wave minimal latency in normal, overweight, and obese patients

To explore the influence of obesity on the F‐wave parameters, we subcategorized the study population into 6 groups of obese controls, nonobese controls, obese with mild CTS, nonobese with mild CTS, obese with moderate CTS, and nonobese with moderate CTS. Table 3 shows the F‐wave parameters across the groups. Three parameters showed significant group differences, and they were as follows: F‐wave chronodispersion (*p* =.040), F‐wave minimal latency (*p* =.036), and median–ulnar minimum F latency difference (*p* =.007). Tukey's post hoc analysis for multiple pairwise comparisons was performed. Table 4 shows the significant pairwise comparison between the groups. Except for median–ulnar minimum F‐latency difference between nonobese, mild CTS versus obese, moderate CTS subjects (*p* =.017), other F‐wave parameters did not distinguish between mild CTS and moderate CTS. However, the differences between the CTS group and the control group were sustained (Table 4). Figure 1 shows median–ulnar *F* (min) latency differences according to BMI in mild and moderate CTS. Figure 2 shows median *F* (min) latency according to BMI in mild and moderate CTS. The significant differences in median *F* (min) latency and MUD F (min) were noticeable between mild and moderate CTS with normal BMI. Of the other F‐wave parameters, the MUD‐*F* (min) was the only index that showed a positive correlation with BMI among the mild CTS group (Supplementary Table 2).

**TABLE 3:** F‐wave parameters among controls and obese and nonobese cohorts with mild and moderate CTS

F‐wave parameter	Persistence (%)	Chronodispersion (ms)	Median minimal F‐wave latency (ms)	F‐M latency (ms)	Minimal ulnar‐median F‐wave latency difference (ms)	Mean duration (ms)	Mean amplitude (uV)	Mean amplitude ratio
Obese controls (*n* = 2)	93.50 ± 9.19	1.05 ± 0.00	29.50 ± 0.56	26.15 ± 0.07	−0.08 ± 2.3	12.32 ± 2.79	5.02 ± 2.36	1.08 ± 0.25
Nonobese, controls (*n* = 20)	96.00 ± 5.79	1.46 ± 0.98	27.43 ± 2.08	24.03 ± 2.03	0.47 ± 4.5	9.27 ± 2.45	5.64 ± 2.78	2.04 ± 0.99
Nonobese, mild CTS (*n* = 20)	87.25 ± 4.72	2.83 ± 0.34	27.15 ± 0.60	23.62 ± 0.59	−0.46 ± 0.51	11.26 ± 0.61	4.17 ± 0.38	2.86 ± 0.55
Obese, mild CTS (*n* = 10)	84.40 ± 6.68	2.26 ± 0.46	26.47 ± 0.85	22.91 ± 0.84	−0.36 ± 0.72	10.55 ± 0.86	4.51 ± 0.53	3.22 ± 0.77
Nonobese, moderate CTS (*n* = 13)	74.23 ± 5.84	2.56 ± 0.43	29.31 ± 0.75	24.57 ± 0.73	1.69 ± 0.63	10.55 ± 0.75	3.58 ± 0.49	2.82 ± 0.68
Obese, moderate CTS(*n* = 26)	84.88 ± 4.13	2.49 ± 0.30	28.67 ± 0.53	23.92 ± 0.52	1.84 ± 0.45	10.57 ± 0.53	4.05 ± 0.33	3.33 ± 0.48
F statistics*	2.286	2.446	2.511	0.888	3.44	1.381	2.260	1.102
P value	0.053	0.040	0.036	0.493	0.007	0.239	0.056	0.365

Note:

One‐way analysis of variance (ANOVA).

**TABLE 4:** Multiple pairwise comparisons with Tukey HSD correction

F‐wave parameters	Between‐group comparisons	P value
F‐wave persistence	Nonobese moderate CTS *Vs* Nonobese control	0.019
Chronodispersion	Nonobese, mild CTS *Vs* Nonobese control	0.035
*F* (min) latency	Nonobese moderate CTS *Vs* Nonobese control	0.051
Median–ulnar difference	Nonobese mild CTS *Vs* Obese moderate CTS	0.017

Abbreviation: CTS, carpal tunnel syndrome.

## 4 DISCUSSION

Our study showed that most F‐wave parameters were significantly different between controls and patients with mild and moderate CTS. Furthermore, when our cohort was categorized based on BMI, F‐wave (min) latency, chronodispersion, and median–ulnar FWLD were the parameters that retained statistically significant differences between the groups. Previous studies of F‐wave parameters in CTS showed that F‐wave parameters, especially *F* (min) latency, *F* (max) latency, and *F* (mean) latency, were significantly prolonged compared with controls. (Sulaiman, 2012) In addition to these parameters, Özge et al. (Özge et al., 2002) found that F‐wave persistence and chronodispersion were delayed considerably in CTS, just like in our study. In their research, Özge et al. showed that F‐wave parameters increased the diagnostic yield and differentiation of CTS pathological subtypes, prominent demyelinating, prominent axonal, and slight demyelinating types (Özge et al., 2002).

Since BMI and height are positively correlated with F‐wave latency in healthy individuals (Huang et al., 2009; Majumdar et al., 2017), we posited that these factors might also play a role in CTS patients. In our cohort, median–ulnar FWLD, chronodispersion, and *F* (min) latency clearly showed a significant difference between obese and nonobese controls and those with mild‐moderate CTS. We found that median–ulnar FWLD was the most distinguishing factor between mild and moderate obese patients. Alemdar in a cohort of 174 hands found that median–ulnar FWLD yielded a higher diagnostic efficacy than median *F* (min) latency on CTS diagnosis. Sander et al (Sander et al., 1999) had earlier demonstrated the utility of median–ulnar FWLDs in the diagnosis of CTS in a study that showed a diagnostic sensitivity of 78%. The authors emphasized its usefulness, particularly in the setting of an underlying concomitant polyneuropathy and anatomical variants. While these findings sounded promising, the study by Mondelli et al. found that most F‐wave parameters generally had low sensitivity in CTS diagnosis. (Mondelli & Aretini, 2015) In the same vein, Uzunkulaoǧlu et al. confirmed that the median–ulnar FWLD could not be a stand‐alone discriminant variable because of its low sensitivity (Uzunkulaoǧlu et al., 2019). Even in studies that demonstrated a diagnostic sensitivity, the sensitivities' modest nature precluded a recommendation of their usage as diagnostic criteria. (Aalemdar, 2015; Mondelli & Aretini, 2015) However, it is yet to be entirely determined if the inclusion of median–ulnar FWLD to existing criteria will increase CTS diagnostic sensitivity in obese individuals. Increased BMI is posited to raise the volume of translocated blood in the upper body (thorax and arms) and potentially lead to fluid volume in the arms and carpal tunnel. (Radecki, 1996) Hence, a causal relationship between local fatty tissue and raised hydrostatic pressure within the carpal tunnel has been suggested. (Werner et al., 1994).

Knowing the predominantly affected types of the median nerve fiber is essential in estimating prognosis and planning early intervention in CTS. Since early motor involvement means more severe CTS, it is worth determining the most initial index of such impairment. However, the analysis of F wave, unlike CMAP, is not as straightforward because the latency and amplitude of F waves fluctuate from response to response since different motor neurons from the pool contribute to consecutive responses. (Mondelli & Aretini, 2015) Our post hoc analysis showed that The *F* (min) wave latency‐a surrogate of the demyelinating process and F‐wave persistence‐indicative of axonal loss, were significantly different between nonobese mild and moderate CTS versus nonobese control. These parameters were of no statistical significance between obese or nonobese mild and moderate CTS. Hence, we can conclude that median nerve demyelination or axonal loss at the carpal tunnel is not significantly different between mild and moderate CTS irrespective of BMI. These findings could be because BMI does not differentiate between muscle and fat accumulation, and between fat locations. (Mondelli, Curti, et al., 2016) Other anthropometric indices like shape index, digit index, hand length/height, wrist–palm ratio, and waist hip‐height ratio, and waist‐stature might better discriminate between fat locations. (Mondelli, Farioli, et al., 2016).

### 4.1 Limitations

Our study is not without its limitations. In addition to its moderate sample size, the controls and those with CTS were not matched for age, gender, and height. A larger, prospective study that will match these variables is advocated.

## 5 CONCLUSION

Although a clear difference exists between F‐wave parameters between asymptomatic controls and those with CTS, F‐wave parameters are not convincingly different between mild and moderate CTS. Median–ulnar FWLD appeared to be a promising discriminant parameter in obese patients with mild and moderate CTS. Overall, F‐wave study is inadequate in distinguishing mild‐moderate CTS even when BMI is considered.

## CONFLICT OF INTEREST

The authors declare no conflicts of interest regarding this work.

## AUTHORS' CONTRIBUTIONS

PBA conceived the idea of the study while REW curated the data. PBA drafted the manuscript. PBA and REW edited and reviewed the manuscript for intellectual content.

### PEER REVIEW

The peer review history for this article is available at https://publons.com/publon/10.1002/brb3.2072.

## Data Availability

The data that support the findings of this study are available from the corresponding author upon reasonable request.
